# Overexpression of PRL7D1 in Leydig Cells Causes Male Reproductive Dysfunction in Mice

**DOI:** 10.3390/ijms17010096

**Published:** 2016-01-13

**Authors:** Yaping Liu, Xingyu Su, Jie Hao, Maoxin Chen, Weijia Liu, Xiaogang Liao, Gang Li

**Affiliations:** 1 Institute of Life Sciences, Chongqing Medical University, Chongqing 400016, China; liuy_p@sina.com (Y.L.); sxyasxy@163.com (X.S.); chenmaoxin1989@163.com (M.C.); weijliu@163.com(W.L.); liaoxg@126.com (X.L.); 2 The First Affiliated Hospital, Chongqing Medical University, Chongqing 400016, China; haojcq@yahoo.com

**Keywords:** *Plr7d1*, male reproductive dysfunction, Leydig cells

## Abstract

Prolactin family 7, subfamily d, member 1 (PRL7D1) is found in mouse placenta. Our recent work showed that PRL7D1 is also present in mouse testis Leydig cells, and the expression of PRL7D1 in the testis exhibits an age-related increase. In the present study, we generated transgenic mice with Leydig cell-specific PRL7D1 overexpression to explore its function during male reproduction. *Prl7d1* male mice exhibited subfertility as reflected by reduced sperm counts and litter sizes. The testes from *Prl7d1* transgenic mice appeared histologically normal, but the frequency of apoptotic germ cells was increased. *Prl7d1* transgenic mice also had lower testosterone concentrations than wild-type mice. Mechanistic studies revealed that *Prl7d1* transgenic mice have defects in the testicular expression of steroidogenic acute regulatory protein (STAR) and hydroxy-delta-5-steroid dehydrogenase, 3 beta- and steroid delta-isomerase cluster (HSD3B). Further studies revealed that PRL7D1 overexpression affected the expression of transferrin (TF) in Sertoli cells. These results suggest that PRL7D1 overexpression could lead to increased germ cell apoptosis and exert an inhibitory effect on testosterone production in Leydig cells by reducing the expression of certain steroidogenic-related genes. In addition, PRL7D1 appears to have important roles in the function of Sertoli cells, which, in turn, affects male fertility. We conclude that the expression level of PRL7D1 is associated with the reproductive function of male mice.

## 1. Introduction

Prolactin (*Prl*) family genes are located on chromosome 13 in mice and chromosome 17 in rats and are expressed mainly in the pituitary gland, uterus and/or placenta [1,2,3]. To date, 26 mouse *Prl* family members have been identified, including prolactin-like proteins, placental lactogens, prolactin family 2, subfamily c, member 2 (*Prl2c2*) and prolactin family 7, subfamily d, member 1 (*Prl7d1*). The majority of these genes are associated with female reproduction [3,4,5]. Based on how diverse signals are transmitted to target cells through the *Prl* receptor or other signaling pathways, *Prl* family members have been divided into classical and non-classical groups.

*Prl7d1*, a non-classical member of the *Prl* family, is more commonly known as proliferin-related protein (*Prp*) and so named because of its close relationship to another *Prl*-related hormone, *Prl2c2* (also known as *Plf*) [6,7]. PRL2C2 is secreted specifically by trophoblast giant cells, and the site of PRL7D1 synthesis has been localized to the basal zone of Day 10 mouse placenta [8]. PRL2C2 is expressed highly in early- to mid-gestation [9], while PRL7D1 is expressed at high levels from mid- to late-gestation [8,10]. PRL7D1 appears to be a potent placental anti-angiogenic hormone, inhibiting endothelial cell migration and generating a barrier zone to limit the growth of maternal and fetal vessels across the placenta [11].

Expression of PRL7D1 was first identified in mouse placenta [6]. However, our previous study showed that PRL7D1 was expressed in both rat and mouse testis Leydig cells. This demonstrated that PRL7D1 was not restricted to placental tissues [12]. Real-time polymerase chain reaction (PCR) and Western blotting results showed that *Prl7d1* mRNA and protein expression during the developmental stage of rat testis increased in an age-dependent manner. In addition, *Prl7d1* silencing did not affect basal testosterone production in the TM3 Leydig cell line, but did attenuate the increase of testosterone production in response to stimulation with human chorionic gonadotropin.

Although *in vitro* studies suggest that PRL7D1 might play roles in male reproduction, its physiological significance *in vivo* requires further study. Because the expression level of PRL7D1 reached its peak in aged testis, we speculated that a high expression level was associated with the age-related decline in male reproductive function. Thus, in the present study, we explored the potential biological activities of PRL7D1 in male reproduction *in vivo* by generating transgenic mice with Leydig cell-specific overexpression of PRL7D1. Male mice overexpressing *Prl7d* exhibited alterations of testosterone secretion and spermatogenesis. This demonstrated that an appropriate level of PRL7D1 expression was critical for the development and function of mouse testis.

## 2. Results

### 2.1. Generation of Prl7d1 Transgenic Mice

**Figure 1:**
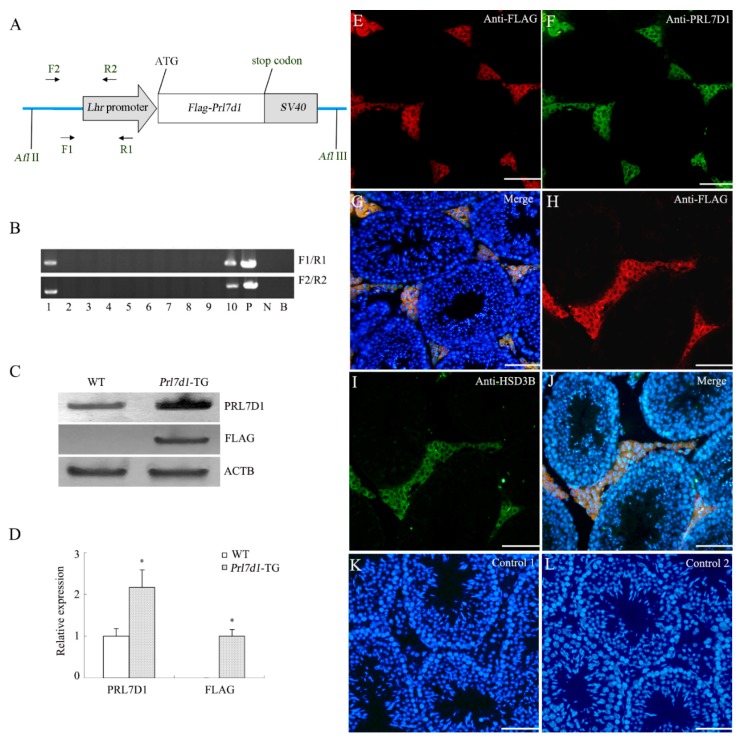
Generation of transgenic mice overexpressing *Prl7d1*. (**A**) Schematic representation of the *Prl7d1* transgenic construct. The *Lhr* promoter was fused to *Flag*-tagged mouse *Prl7d1* cDNA and *SV40* polyadenylase signal. The FLAG epitope (DYKDDDDK) was introduced into the amino-terminus of the PRL7D1 protein. The primers used for the detection of the transgene are indicated by arrows; (**B**) PCR analysis of the genomic integration of the mouse *Prl7d1* transgene. N, negative control; P, positive control (*Prl7d1* transgenic construct); B, blank control; (**C**) Western blot analyses of PRL7D1, FLAG and actin beta (ACTB) in the testes of four-month-old *Prl7d1* transgenic (*Prl7d1*-TG) and wild-type (WT) mice; (**D**) The relative expression of PRL7D1 and the FLAG-tagged protein in the testes of mice are shown following their normalization against ACTB. Data are expressed as the means ± SD (*n* = 6). The protein level within the WT group was set to one. * *p* < 0.01 compared to the WT group; (**E**–**L**) Double immunofluorescence staining revealed that FLAG-tagged protein (red fluorescence) co-localized with PRL7D1 (green fluorescence) (**E**–**G**) or HSD3B (green fluorescence) (**H**–**J**) in Leydig cells of adult *Prl7d1* mouse testes. Control 1: testicular tissue sections were incubated with IgG1; Control 2: immunofluorescent staining of FLAG-tagged protein in WT testis sections. There was no positive staining in Leydig cells. Staining with DAPI (blue fluorescence) was used to label the nuclei of individual cells found in the section. The scale bar is 50 μm.

In males, the luteinizing hormone receptor (*Lhr*), located on the surface of Leydig cells of testis, plays a crucial role in the regulation of testosterone production [13,14,15]. Additionally, *Lhr* is also expressed in several non-gonadal tissues [13,16,17,18]. A fragment of the *Lhr* upstream promoter had been previously validated to be sufficient for the expression of transgene in Leydig cells of testis [19,20]. Thus, to study the effect of PRL7D1 overexpression within testis, we generated transgenic mice carrying the *Flag-Prl7d1*-*SV40* transgene under control of the *Lhr* promoter (Figure 1A) [19,20]. Using PCR genotyping, we identified two male founder mice (Lines 1 and 10) that were positive for the mouse *Prl7d1* transgene (Figure 1B). Additionally, the direct sequencing of the PCR fragment confirmed the presence of transgene (data not shown). Expression levels of PRL7D1, evaluated by Western blot analyses of testicular lysates from four-month-old transgenic mice derived from Line 1 founder mice revealed promoter activation in the testis with PRL7D1 upregulation as compared to wild-type mice (Figure 1C,D). There were no apparent differences in the levels of testicular PRL7D1 between the two transgenic lines (data not shown), and Line 1 mice were used for all subsequent studies.

To confirm the expression of the *Prl7d1* transgene, the FLAG-epitope protein was detected by Western blotting in the transgenic mouse testes at a molecular weight appropriate for PRL7D1, but not in the wild-type littermates (Figure 1C,D). Immunofluorescent analyses revealed that FLAG-tagged PRL7D1 co-localized with PRL7D1 (Figure 1E–G) or HSD3B (a marker of Leydig cells) (Figure 1H–J) in Leydig cells from sections of transgenic testicular tissue. These findings further substantiated the expression of the transgenic construct within the testes.

### 2.2. Body and Testicular Weights

No abnormal behavioral characteristics or anatomical changes were noted in either wild-type or transgenic mice. Body weights did not differ significantly between wild-type (29.2 ± 2.64 g) and *Prl7d1* (28.3 ± 2.07 g) mice (*n* = 6). In addition, testes and epididymides weights were not significantly different between wild-type and transgenic mice (0.1 ± 0.007 g *versus* 0.088 ± 0.011 g and 0.041 ± 0.004 g *versus* 0.039 ± 0.006 g, respectively).

### 2.3. Effects of Overexpressing Prl7d1 on Sperm Count and Fertility in Prl7d1 Transgenic Mice

The cauda epididymal sperm number of *Prl7d1* transgenic mice was markedly lower than that of wild-type mice (*p* < 0.01). The presence of a vaginal plug was observed in all wild-type females mated with wild-type or *Prl7d1* transgenic mice. However, only five of 12 females became pregnant after mating with *Prl7d1* males, whereas 11 of 12 females were pregnant after mating with wild-type males. Furthermore, the litter size resulting from wild-type females mating with *Prl7d1* males was significantly smaller (*p* < 0.01) (Table 1). Together, these results suggested that *Prl7d1* males exhibited diminished fertility.

**Table 1:** Fertility and sperm counts of 4-month-old *Prl7d1* transgenic (*Prl7d1-*TG) and wild-type (WT) mice (*n* = 6).

	WT	*Prl7d1-*TG
Pregnant females (*n* = 12)	11	5 ^#^
Litter size	7.2 ± 0.9	4.2 ± 1.3 *
sperm count (×10^6^)	13.1 ± 1.5	7.5 ± 1.4 *

^#^ *p* < 0.05, * *p* < 0.01 compared to the WT group.

### 2.4. Histology of Prl7d1 Transgenic Mice Testes

**Figure 2:**
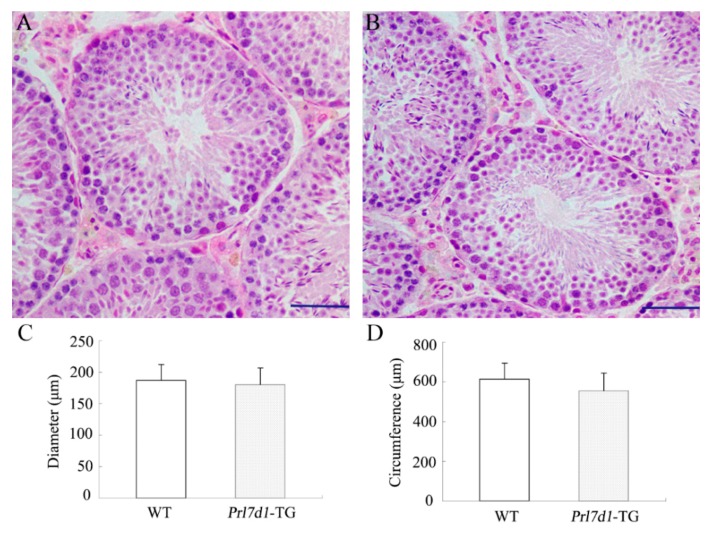
Histological analyses of testis from four-month-old wild-type (WT) (**A**) and *Prl7d1* transgenic (*Prl7d1*-TG) mice (**B**). Sections were stained with hematoxylin and eosin. The scale bar is 50 μm; (**C**) The diameter of the seminiferous tubules and (**D**) the circumference of the seminiferous tubules were normal in *Prl7d1* transgenic mice. Results for each measurement are expressed as the means ± SD from a minimum of 60 randomly-selected seminiferous tubules from six mice of each phenotype.

To determine whether the overexpression of PRL7D1 was associated with defects in testis architecture, testis sections from wild-type and transgenic mice were examined histologically. Testes from both groups appeared histologically normal and showed a highly integrated seminiferous tubule structure (Figure 2A,B). Additionally, the diameter and circumference of seminiferous tubules were similar between *Prl7d1* transgenic and wild-type mice (Figure 2C,D).

### 2.5. Overexpression of Prl7d1 in Leydig Cells Increases Germ Cell Apoptosis and Upregulates Cleaved Caspase 3 (17 kDa) and BCL2-Associated X Protein Expression in Testes

**Figure 3:**
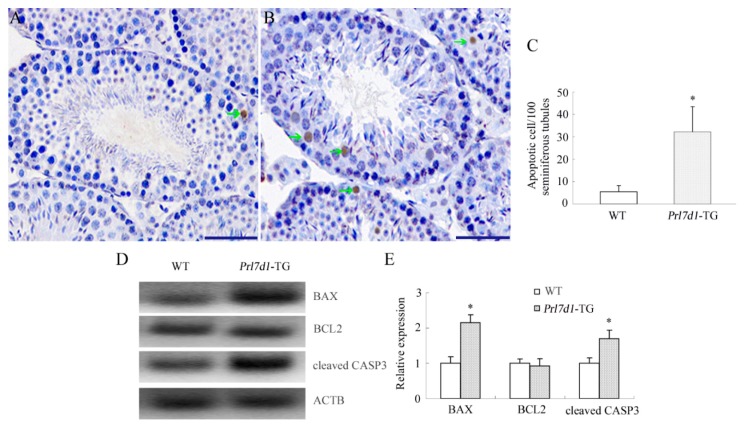
PRL7D1 overexpression results in increased germ cell apoptosis and upregulated expression of cleaved CASP3 and BAX in testes. Representative microphotographs of the TUNEL assay performed in testis sections from wild-type (WT) (**A**) and *Prl7d1* transgenic (*Prl7d1*-TG) (**B**) mice. TUNEL-positive cells are stained brown (green arrows). The scale bar is 50 μm; (**C**) Quantitative assessment of the TUNEL-positive cell number per 100 tubules/testis (*n* = 6); (**D**,**E**) Protein levels of BAX, BCL2 and cleaved CASP3 in testes were detected by Western blot analyses. The relative expression levels of the proteins were determined using densitometric analyses of the data that were normalized against the ACTB signal (*n* = 6). The protein level in the WT group was set to one. Each bar represents the mean ± SD. * *p* < 0.01 compared to the WT group.

In the adult testis, apoptosis plays a major role in removing damaged germ cells from the seminiferous epithelium for maintaining functional spermatogenesis. Various physiological (such as deprivation of intratesticular testosterone) and environmental factors can trigger germ cell apoptosis, and increased germ cell apoptosis has been linked to a variety of adverse reproductive outcomes, including impaired fertility [21,22]. To examine the potential mechanisms underlying diminished fertility in *Prl7d1* male mice, we next evaluated the impact of the *Prl7d1* transgene on germ cell apoptosis using the terminal deoxynucleotidyl transferase dUTP nick-end labeling (TUNEL) assay. We observed more apoptotic cells in *Prl7d1* than in wild-type tubules (Figure 3A–C), and the apoptotic cells were mostly pachytene spermatocytes and round spermatids.

Previous studies suggested the involvement of pro- and anti-apoptotic proteins in regulating germ cell apoptosis [23,24]. Western blot analyses showed that the expression of pro-apoptotic cleaved CASP3 and BAX, but not anti-apoptotic B cell leukemia/lymphoma 2 (BCL2), were upregulated in the *Prl7d1* testis (Figure 3D–E). This indicated that an imbalance of pro-apoptotic/anti-apoptotic factors was associated with the increase in germ cell apoptosis induced by PRL7D1 overexpression.

### 2.6. Decreased Levels of Serum and Intra-Testicular Testosterone, but Not Gonadotropin-Releasing Hormone and Luteinizing Hormone, in Prl7d1 Transgenic Mice

**Figure 4:**
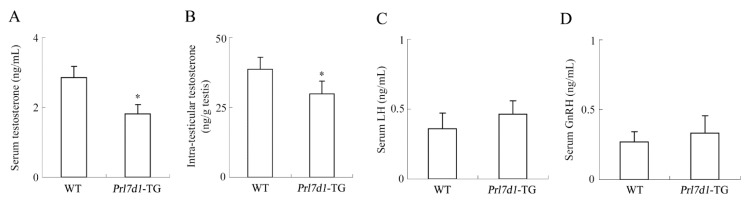
The levels of serum testosterone (**A**), LH (**C**) and GnRH (**D**) and intra-testicular testosterone (**B**) in four-month-old *Prl7d1* transgenic (*Prl7d1*-TG) and wild-type (WT) mice. Testosterone, LH and GnRH levels were measured by ELISA. Data are expressed as the means ± SD (*n* = 6). * *p* < 0.01 compared to the WT group.

ELISA analyses showed that the levels of serum testosterone were significantly decreased in *Prl7d1* transgenic mice (*p* < 0.01) (Figure 4A). To explore whether the decline in serum testosterone was due to decreased testicular production, we determined the testosterone levels in testes from wild-type and *Prl7d1* transgenic mice. Consistent with serum testosterone levels, intra-testicular testosterone levels were also significantly reduced in *Prl7d1* transgenic mice compared to those in wild-type mice (*p* < 0.01) (Figure 4B). These findings indicated that PRL7D1 overexpression inhibited testosterone production.

The hypothalamic-pituitary-gonadal (HPG) axis plays important roles in regulating the male reproductive functions. GnRH, a hormone produced by specific neurons in the hypothalamus, triggers the release of LH from the pituitary gland. LH can increase testosterone production by affecting Leydig cells in the testes [25,26]. To further investigate whether reduced testosterone in *Prl7d1* transgenic mice was associated with the disruption of GnRH and LH levels, serum GnRH and LH levels were analyzed by ELISA. As shown in Figure 4C,D, there were no significant differences of serum GnRH and LH levels between the wild-type and *Prl7d1* transgenic mice, which demonstrated that PRL7D1 overexpression exerted its inhibitory effect on testosterone synthesis not through directly affecting GnRH or LH levels.

### 2.7. Overexpression of Prl7d1 in Leydig Cells Decreases the Expression of STAR and HSD3B

**Figure 5:**
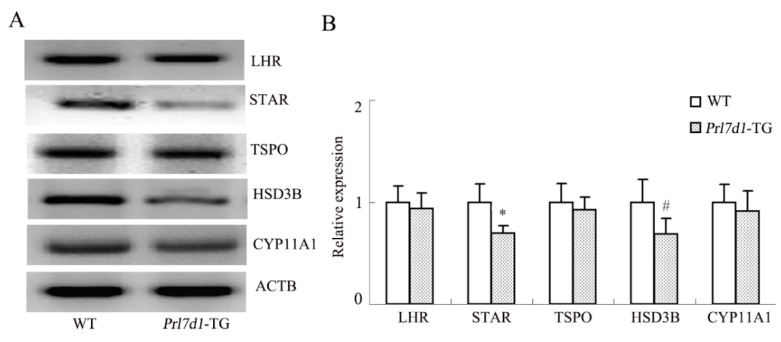
Western blot analyses of the LHR, STAR, TSPO, HSD3B and CYP11A1 in the testes of wild-type (WT) and *Prl7d1* transgenic (*Prl7d1*-TG) mice. (**A**) Representative Western immunoblots; (**B**) The relative expression levels of the proteins were determined using densitometric analyses of the data that were normalized against the ACTB signal (*n* = 6). The protein level in the control group was set to one. Each bar represents the mean ± SD. ^#^ *p* < 0.05, * *p* < 0.01 compared to the WT group.

Potential molecular mechanisms underlying the PRL7D1-mediated decrease in testosterone production in Leydig cells were investigated by analyzing the protein levels of the LHR and several enzymes involved in the conversion of cholesterol to testosterone. Western blot analyses showed that the expression of STAR and HSD3B was decreased by PRL7D1 overexpression, while no significant difference was observed in the expression of the LHR, translocator protein (TSPO) or cytochrome P450, family 11, subfamily A, polypeptide 1 (CYP11A1) (Figure 5).

### 2.8. Overexpression of Prl7d1 in Leydig Cells Decreases TF Expression

**Figure 6:**
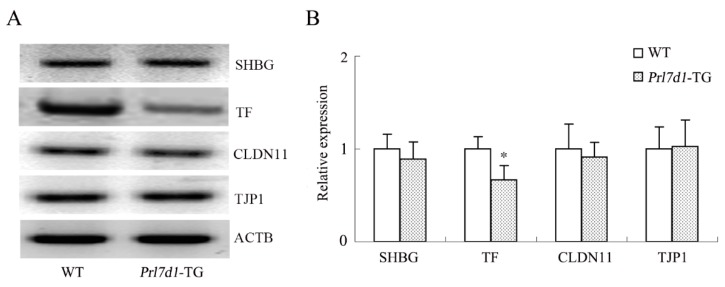
Western blot analyses of the SHBG, TF, CLDN11 and TJP1 in the testes of wild-type (WT) and *Prl7d1* transgenic (*Prl7d1*-TG) mice. (**A**) Representative western immunoblots; (**B**) The relative expression levels of the proteins were determined using densitometric analyses of the data that were normalized against the ACTB signal (*n* = 6). The protein level in the control group was set to one. Each bar represents the mean ± SD. * *p* < 0.01 compared to the WT group.

One of the major functions of Sertoli cells is to produce molecules important in spermatogenesis. To determine whether PRL7D1 overexpression affected the function of Sertoli cells, we compared the expression levels of several proteins expressed specifically in Sertoli cells. There were no significant changes in the expression of sex hormone-binding globulin (SHBG), tight junction protein 1 (TJP1) and claudin-11 (CLDN11) in testis lysates between wild-type and *Prl7d1* transgenic mice. However, TF levels were significantly downregulated in *Prl7d1* transgenic mice testis (*p* < 0.01) (Figure 6), suggesting that PRL7D1 affected TF expression.

## 3. Discussion

Previous studies demonstrated that testes have two functions: steroidogenesis (the biosynthesis of steroid hormones) and spermatogenesis (the production of spermatozoa) [21,25]. These two complex functions of testes are highly regulated by multiple factors, such as hormones, proteases, protease inhibitors, signaling molecules, growth factors and cell adhesion molecules, all of which are produced by various cell types within the testes [27]. Thus, exploring the roles of these molecules is important to understand testicular development and function.

Identifying key molecules that contribute to testis function often involves the use of genetically-modified (transgenic and knockout) mouse models. For example, mice that overexpress insulin [28], *Bcl2* [29], spermatogenesis associated 17 [30] or SRY (sex determining region Y)-box 8- [31] and basigin-null [32] male mice exhibit alterations in testosterone secretion and/or spermatogenesis. These effects suggest that these molecules play important roles in male reproduction.

To our knowledge, this is the first mouse model altering the expression of PRL7D1 that leads to clear changes in the reproductive capacity of males. Because the expression of PRL7D1 in testes of rats increases with age [12], and to better understand the physiological role of PRL7D1 in male reproduction, we generated gain-of-function *Prl7d1* transgenic mice using the *Lhr* promoter. This promoter is sufficient to initiate and maintain the Leydig cell-specific expression of nuclear receptor subfamily 0, group B, member 1 [20] and β-galactosidase [19], and our results suggest that *Lhr* is also an appropriate promoter for studying the overexpression of PRL7D1 in Leydig cells.

*Prl7d1* transgenic mice were viable and developed normally to adulthood. The adult body, testes and epididymides weights were not impacted by overexpressing the *Prl7d1* transgene. However, the average litter size and sperm counts were significantly decreased in transgenic males when compared to the control wild-type males. This suggested that overexpression of the *Prl7d1* transgene decreased male fertility. Histological evaluation of *Prl7d1* mouse testes showed that overexpressing PRL7D1 did not significantly affect their morphological development. However, a significant increase of apoptotic germ cells was detectable in the seminiferous tubule of *Prl7d1* transgenic mice compared to wild-type mice. By Western blot analyses, cleaved CASP3 and BAX, but not BCL2, were significantly increased in *Prl7d1* mouse testes. Thus, upregulated expression of pro-apoptosis-related proteins may, at least in part, account for the increased frequency of apoptotic germ cells in seminiferous tubules and eventually result in decreased male fertility in *Prl7d1* transgenic mice.

Testosterone is essential to maintain normal testicular development and spermatogenesis, as well as secondary sexual characteristics. In testis, testosterone is produced by Leydig cells under the regulation of LH signaling [33]. Serum and intra-testicular testosterone levels were significantly decreased in *Prl7d1* transgenic mice compared to wild-type mice. This suggested that PRL7D1 plays a negative role in testosterone production.

Decreased testosterone levels are thought to arise from disturbed steroidogenesis in Leydig cells or reduced hypothalamic GnRH or pituitary LH stimuli [25,26]. Serum GnRH and LH levels were unchanged in transgenic mice, indicating that PRL7D1 overexpression had no apparent effect on the GnRH and LH levels, and the reduced testosterone production was not through alterations in GnRH or LH signaling. Thus, we speculated that defects in the steroidogenic pathway were responsible for decreased testosterone in *Prl7d1* transgenic mice.

The capacity of Leydig cells to synthesize testosterone is highly related to the LHR and steroidogenic enzymes [34]. Binding of LH to its receptor triggers various testosterone biosynthetic events. These begin with the transfer of cholesterol from the outer to the inner mitochondrial membrane through the transduceosome, a complex composed of cytosolic proteins that include STAR [35,36] and the outer mitochondrial membrane protein TSPO [37,38]. After translocation to the inner mitochondrial membrane, cholesterol is converted to pregnenolone and eventually testosterone through several enzymatic steps involving the enzymes CYP11A1 and HSD3B. In recent years, increasing evidence showed that many factors that diminish testosterone production in male testis, including aging and exposure to environmental toxicants, could be explained by reductions in LHR and/or steroidogenic enzymes [33,39,40,41,42]. In the present study, protein levels of LHR, TSPO and CYP11A1 in *Prl7d1* transgenic mice were not significantly different from controls. However, STAR and HSD3B were significantly decreased, indicating that changes in these factors may explain how PRL7D1 overexpression inhibits testosterone synthesis.

In mammalian testes, Sertoli cells, the primary supportive cells of the seminiferous tubules, interact directly with germ cells to control their proliferation and differentiation [43]. Thus, adequate Sertoli cell function is essential for spermatogenesis. Factors produced by Leydig cells have direct effects on Sertoli cells, stimulating their proliferation and the expression of several factors that may contribute to spermatogenesis [44,45,46]. To determine whether PRL7D1 affects Sertoli cell function, we assessed the expression of CLDN11, TJP1, TF and SHBG, proteins that are expressed specifically in Sertoli cells. Our results demonstrated that PRL7D1 overexpression did not affect the expression of CLDN11, TJP1 or SHBG. However, TF expression was decreased in *Prl7d1* transgenic mice. TF plays an important role in spermatogenesis by transporting iron to spermatogenic cells and is useful as a marker for Sertoli cell function [47,48,49]. The lower expression level of TF in *Prl7d1* transgenic mice implied that Sertoli cell function was impaired [48,49]. These results suggested that increased PRL7D1 expression may regulate the function of Sertoli cells. In addition, TF expression in Sertoli cells is controlled by androgen signaling [50], then the reduction in TF may be caused by the reduction in intratesticular testosterone seen in *Prl7d1* transgenic mice. Thus, PRL7D1 overexpression may also play an indirect role in Sertoli cell functions.

In summary, our results provide *in vivo* evidence showing that PRL7D1 overexpression in Leydig cells affects the reproductive functions of male mice. Decreased testosterone production in Leydig cells, increased apoptotic germ cells in seminiferous tubules and reduced expression of TF in Sertoli cells may, at least partly, account for the decreased fertility in *Prl7d1* transgenic mice. However, *Lhr* is also expressed in several extra gonadal sites [16,51], then the *Lhr* promoter may drive *Prl7d1* transgene expression on other male organs, and fetuses with the *Prl7d1* transgene may be more susceptible to death during embryonic development. Therefore, the possible effects of *Prl7d1* expression on other organs driven by *Lhr* promoter need to be investigated further.

## 4. Experimental Section

### 4.1. Materials

Protein extraction kits and Beyo ECL Plus Western blotting reagents were purchased from Beyotime Biotechnology (Jiangsu, China). ELISA kits for testosterone, GnRH and LH were purchased from USCN (Wuhan, China). The pMD18-T vector was purchased from Takara (Dalian, China). The remaining chemicals were purchased from Sangon Biotech (Shanghai, China).

### 4.2. Transgene Construction

Transgenic mice with Leydig cell-specific overexpression of PRL7D1 were generated using a fragment of the *Lhr* upstream promoter. The transgenic construct contained the following components: the upstream promoter region of the *Lhr*, *Flag* tags, the complete open reading frame of mouse *Prl7d1* and the simian virus 40 (*SV40*) polyadenylation sequence. The *Lhr* promoter fragment was amplified from C57BL/6 mice genomic DNA using the primers 5′-TGTGCGGCAAGGCATCTAT-3′ and 5′-TGATGCCGTCTTCCTTTTC-3′. To clone *Prl7d1* from the C57BL/6 mice and add the FLAG epitope (DYKDDDDK) to the PRL7D1 protein, the primers 5′-TGGCGGGCCATGGACTACAAGGACGACGATGACAAGCTCCCTTCTTTGAT-3′ and 5′-TGATCATTACTTATCTAGATCAAAATTCAGAGTAG-3′ were used. The *SV40* fragment was amplified from the pcDNA3.1 vector using the primers 5′-GAATTTTGATCTAGATAAGTAATGATCATAATC-3′ and 5′-GATCCTCTGGAGATACAGACATGATAAGATACAT-3′. Subsequently, the *Flag*-*Prl7d1*-*SV40* fragment was generated by in-fusion PCR using the primers 5′-TGGCGGGCCATGGACTACAAGGACGACGATGACAAGCTCCCTTCTTTGAT-3′ and 5′-GATCCTCTGGAGATACAGACATGATAAGATACAT-3′. The transgenic cassette, *Lhr-Flag*-*Prl7d1*-*SV40*, was generated by in-fusion PCR using the primers 5′-ATAATCAATGTCAACCATTCCAACCTTTCTCCTACA-3′ and 5′-GATCCTCTGGAGATACAGACATGATAAGATACAT-3′. The resultant fragment was cloned into the pMD18-T-EGFP vector for sequencing.

### 4.3. Generation of Prl7d1 Overexpressing Mice

C57BL/6 mice were obtained from the Experimental Animal Center at Chongqing Medical University. Purified transgenic constructs were microinjected into the pronuclei of fertilized oocytes from these mice. To identify founder (F0) transgenic mice, tail biopsies were collected from two-week-old mice for the isolation of genomic DNA using the Universal Genomic DNA Extraction Kit (Takara, Dalian, China). The resulting genomic DNA samples were screened by PCR using the primers F1 (5′-CAAGTGGGCAGTTTACC-3′), R1 (5′-GTACTGAGTGGCTCGTCA-3′), F2 (5′-TCAAACCGCTATCCACG-3′) and R2 (5′-TTTGTCCCGAGCCATCC-3′). The amplified products were resolved using electrophoresis in 1.5% agarose gels.

Animals were maintained on a 12-h light/dark cycle with free access to food and water. All experiments comparing wild-type and *Prl7d1* transgenic mice used littermates or age-matched animals derived from the same breeding colony. All animal studies were approved by the Animal Care and Use Committee of Chongqing Medical University.

### 4.4. Blood Collection and Tissue Removal

Blood and tissue samples were collected between 9:00 and 10:30 a.m. Briefly, mice were anesthetized with 6% chloral hydrate and exsanguinated by closed cardiac puncture. Sera were collected and stored frozen at −80 °C until further use. Testes and epididymides were removed rapidly and weighed. Tissues were fixed with formalin for routine histology or kept frozen at −80 °C for subsequent analyses.

### 4.5. Histopathological Examination

Formalin-fixed testes were embedded in paraffin, and 4-μm sections were cut. The sections were de-waxed and then re-hydrated through a descending series of alcohol concentrations to distilled water. Sections were stained with hematoxylin and eosin. The samples were analyzed for changes in testicular morphology and structure using light microscopy. Assessment of the diameter and circumference of seminiferous tubules was conducted using ImageJ software (National Institutes of Health, Bethesda, MD, USA). Round or nearly round tubules were chosen randomly for this measurement according to the protocol developed by Neves *et al.* [52].

### 4.6. In Situ Detection of Apoptosis

Apoptosis was evaluated using the TUNEL Apoptosis Assay Kit (Beyotime Biotechnology) following the manufacturer’s instructions. Briefly, testis sections were incubated in proteinase K solution (20 g/mL) for 15 min and rinsed in phosphate-buffered saline. After endogenous peroxidase activity was inhibited using a 3% H_2_O_2_ solution, sections were incubated with 50 μL of a biotin-labeled solution at 37 °C for 60 min. Following washing with phosphate-buffered saline, 50 μL of a streptavidin-HRP working solution were added for 30 min at room temperature. Tissue sections were treated with 3,3′-diaminobenzidine tetrahydrochloride and counterstained with hematoxylin. Finally, the sections were observed under a light microscope. At least 50 tubular cross-sections were selected randomly from each section, and two sections from each animal were examined as described by Kim *et al.* [53].

### 4.7. Immunofluorescent Detection of FLAG-Tagged PRL7D1 Protein

To verify whether FLAG-tagged PRL7D1 was expressed in Leydig cells, the cellular co-localization of FLAG with PRL7D1 or HSD3B was examined in mouse testes using a double immunofluorescence staining. Briefly, sections were stained overnight with a mixture of rabbit anti-FLAG (1:100) IgG together with rat anti-PRL7D1 (1:100) or goat anti-HSD3B IgG (1:100). Slides were washed with phosphate-buffered saline and then incubated with a mixture of tetramethylrhodamine-conjugated anti-rabbit IgG and fluorescein isothiocyanate-conjugated anti-rat or anti-goat IgG for 1 h. Fluorescent images were obtained using a fluorescence microscope.

### 4.8. Sperm Counts

Spermatogenesis was determined by counting the cauda epididymal sperm. Epididymides were minced in Dulbecco’s Modified Eagle Medium to release the sperm. Sperm were counted in a hemocytometer under a light microscope.

### 4.9. LH, GnRH and Testosterone Measurements

Serum testosterone, LH and GnRH levels and intratesticular testosterone levels from testis homogenates were measured using an ELISA kit according to the manufacturer’s instructions. The testosterone, LH or GnRH value of each sample was extrapolated from a standard curve prepared with known concentrations of testosterone, LH or GnRH.

### 4.10. Assessment of Fertility

To assess male fertility, two wild-type female mice were introduced per male. Mated females were checked daily for vaginal plugs, which is a marker of successful mating. After eight days, the male was removed and housed separately. Females were checked for pregnancy, and the number of offspring per pregnant female was recorded.

### 4.11. Western Blotting

The right testis was removed from each animal for protein extraction using radioimmunoprecipitation assay lysis buffer. Protein concentrations were determined using the bicinchoninic acid assay. Proteins were separated on 10% sodium dodecyl sulfate-polyacrylamide gels and transferred onto polyvinylidene difluoride membranes. The membranes were then hybridized with diluted polyclonal antibodies (Table 2). Each membrane was washed in TBS containing 0.1% Tween 20 and incubated with the corresponding secondary antibody. Specific proteins were detected using the Beyo-ECL Kit following the manufacturer’s protocol. The values were normalized against ACTB, which served as the loading control.

**Table 2:** Antibodies used in Western blotting.

Antibody	Dilutions	Host Species	Catalog Number	Vendor
SHBG	1:200	Rabbit	bs-2410R	Bioss
BAX	1:200	Rabbit	bs-0127R	Bioss
BCL2	1:200	Rabbit	bs-0032R	Bioss
CASP3	1:200	Rabbit	bs-0081R	Bioss
CYP11A1	1:200	Rabbit	bs-10099R	Bioss
TSPO	1:200	Rabbit	bs-3674R	Bioss
CLDN11	1:300	Rabbit	36-4500	Life Technologies
TJP1	1:300	Rabbit	61-7300	Life Technologies
FLAG	1:1000	Rabbit	20543-1-AP	Proteintech
TF	1:500	Rabbit	17435-1-AP	Proteintech
LHR	1:500	Rabbit	19968-1-AP	Proteintech
HSD3B	1:300	Goat	sc-30821	Santa Cruz Biotechnology
STAR	1:300	Rabbit	sc-25806	Santa Cruz Biotechnology
PRL7D1	1:300	Rabbit	sc-98474	Santa Cruz Biotechnology

### 4.12. Statistical Analyses

Data are expressed as the means ± standard deviations. Significant differences were determined by Student’s *t*-test. Fertility rates in adult animals from the two groups were compared using Fisher’s exact test. *p*-values less than 0.05 were considered statistically significant.

## 5. Conclusions

In the current study, we generated mice overexpressing *Prl7d1* and found that increasing the expression of this protein decreases male fertility and testicular steroidogenesis by reducing certain steroidogenic-related proteins. Importantly, our findings suggest that PRL7D1 is most likely involved in Sertoli cell functions that are critical for normal spermatogenesis. Thus, we speculate that the increased expression of PRL7D1 plays a negative role in reproductive function.
